# Acute Cartilage Injury Induced by Trans-Articular Sutures

**DOI:** 10.1177/19476035211029704

**Published:** 2021-07-08

**Authors:** Matic Ciglič, Tomaž Marš, Mitja Maružin, Armin Alibegović, Miha Vesel, Matej Drobnič

**Affiliations:** 1 Department of Traumatology, University Medical Centre Ljubljana, Ljubljana, Slovenia; 2 Institute for Pathologic-Physiology, Faculty of Medicine, University of Ljubljana, Ljubljana, Slovenia; 3 Institute of Forensic Medicine, Faculty of Medicine, University of Ljubljana, Ljubljana, Slovenia; 4 Department of Radiology, University Medical Centre Ljubljana, Ljubljana, Slovenia; 5 Department of Orthopedic Surgery, University Medical Centre Ljubljana, Ljubljana, Slovenia; 6 Chair of Orthopedics, Faculty of Medicine, University of Ljubljana, Ljubljana, Slovenia

**Keywords:** articular cartilage, suture, polydioxanone, polyglactin, live/dead staining

## Abstract

**Objective:**

To determine the extent of acute cartilage injury by using trans-articular sutures.

**Methods:**

Five different absorbable sutures, monofilament polydioxanone (PDS) and braided polyglactin (Vicryl), were compared on viable human osteochondral explants. An atraumatic needle with 30 cm of thread was advanced through the cartilage with the final thread left in the tissue. A representative 300 μm transversal slice from the cartilage midportion was stained with Live/Dead probes, scanned under the confocal laser microscope, and analyzed for the diameters of (a) central “Black zone” without any cells, representing *in situ* thread thickness and (b) “Green zone,” including the closest Live cells, representing the maximum injury to the tissue. The exact diameters of suture needles and threads were separately measured under an optical microscope.

**Results:**

The diameters of the Black (from 144 to 219 µm) and the Green zones (from 282 to 487 µm) varied between the different sutures (*P* < 0.001). The Green/Black zone ratio remained relatively constant (from 1.9 to 2.2; *P* = 0.767). A positive correlation between thread diameters and PDS suturing material, toward the Black and Green zone, was established, but needle diameters did not reveal any influence on the zones.

**Conclusions:**

The width of acute cartilage injury induced by the trans-articular sutures is about twice the thread thickness inside of the tissue. Less compressible monofilament PDS induced wider tissue injury in comparison to a softer braided Vicryl. Needle diameter did not correlate to the extent of acute cartilage injury.

## Introduction

Articular cartilage lesions are common joint pathology and remain a challenge for surgeons despite the evolution of techniques for their treatment.^1-3^ Trans-articular sutures were introduced to the field of cartilage repair with the classical autologous chondrocyte implantation for the fixation of periosteal cover.^4-11^ Their use later expanded to application of synthetic membrane coverages.^11-16^ Today the use of a 3-dimensional cartilage repair scaffolds, which rely on press-fit technique or fibrin glue, is leveraged to limit their use, but trans-articular sutures provide additional stability that cannot be entirely replicated by other means.^17^ Trans-articular sutures may also be used for the re-fixation of cartilage flaps or thin osteochondral fragments,^18,19^ and for the retention of the trochlear articular surface during certain types of trochleoplasties.^20^ Their use has also been reported in the fields of ENT and plastic surgery.^21-24^

The use of trans-articular sutures should be as nontraumatic as possible. In particular, it should induce minimal inflammatory response and foreign body reactions. However, the sutures also need to be robust enough to allow penetration through the relatively resistant tissue and have enough tissue holding capacity to prevent detachment or migration of the biomaterial or transplant.^21,22^

Sutures are usually passed with an inside-out suturing technique: entering the cartilage on the vertical side facing the lesion and exiting on the articular cartilage surface.^25^ Most commonly, thin absorbable materials are used (4-0 to 6-0), while the needle design (either oval or triangular) and thread material (either monofilament polydioxanone or braided polyglactin) are mostly dependent on the surgeon’s preference. Only limited reports from animal studies are available on the possible negative effects of the use of trans-articular sutures leading to a localized cartilage degeneration around the suture channel.^26^

The aim of the study was to determine the best commercially available needle and suture combination that would result in the smallest possible acute cartilage injury resulting from the trans-articular passage. Based on previous studies, a combination of viable osteochondral cylinders procured post-mortem, Live/Dead staining, and analysis under a confocal laser microscope represents a competent *ex vivo* model for such a test.^27-31^ We hypothesized that the zone of cartilage death is influenced by the needle and thread diameters, and also by the mechanical properties of suturing materials.

## Methods

The study protocol followed the requirements of the ethical approval issued by the National Medical Ethics Committee of the Republic of Slovenia (No. 74/12/01).

### Osteochondral Cylinders

Forty osteochondral cylinders (Ø 6 mm, depth 10 mm) were procured from femoral condyles and trochleas of adult male donors during autopsies. During the procedure mosaicplasty coring instruments (Helipro, Lesce, Slovenia) were used without drilling. All the donors had died of a sudden traumatic event less than 48 hours prior to the procurement. The procured knee joints were not exposed to a direct trauma and the procured cartilage surfaces showed no macroscopic signs of degeneration—International Cartilage Repair Society grade 0.^32^ The donors had no medical history of a systemic disease that could result in cartilage deterioration. The cylinders were procured under aseptic conditions, immediately transferred into the Dulbecco’s modigied Eagle medium supplemented with penicillin (50 units/mL) and streptomycin (50 mg/mL), and stored at 4 °C until further manipulation.

### Trans-Articular Sutures

The experiment was conducted the following day at room temperature. The cylinders were used two at a time. Eight testing repetitions were performed for each suture. A new suture package was opened and moistened with a lactated Ringer’s solution each time. All sutures were performed by a single surgeon experienced in the autologous chondrocyte implantation. First, the bony part of the cylinder was clamped into a metal holder. Then, an atraumatic needle with 30 cm of absorbable thread was advanced through the cartilaginous part in an inside-out direction using the needle holder and a free-hand technique. The needle entry point was on the side of the cartilaginous cylinder and the exit point at the center of the articular surface—imitating the inside-out technique of periosteum suturing. The final 1 cm of the thread remained in the cartilage tissue for further image evaluation.

Five different commercially available combinations of needles and threads were used (all products by Ethicon, Johnson & Johnson International):

1. PDS II RB-1 (polydioxanone) violet monofilament absorbable 4-0 suture with semicircular 17 mm long round bodied taper point needle with industrially given needle diameter 18 mil (457 µm)
2. PDS II RB-2 (polydioxanone) violet monofilament absorbable 5-0 suture with semicircular 13 mm long round bodied taper point needle with industrially given needle diameter 16 mil (406 µm)
3. Coated Vicryl RB-1 Plus (polyglactin 910) violet braided absorbable 4-0 suture with semicircular 17 mm long round bodied taper point flat needle with industrially given needle diameter 18 mil (457 µm)
4. Coated Vicryl RB-1 Plus (polyglactin 910) violet braided absorbable 5-0 suture with semicircular 17 mm long round bodied taper point flat needle with industrially given needle diameter 18 mil (457 µm)
5. Coated Vicryl RB-2 (polyglactin 910) undyed braided absorbable 5-0 suture with semicircular 13 mm long round bodied taper point needle with industrially given needle diameter 12 mil (305 µm)^33^

### Sample Staining

**Figure 1.**
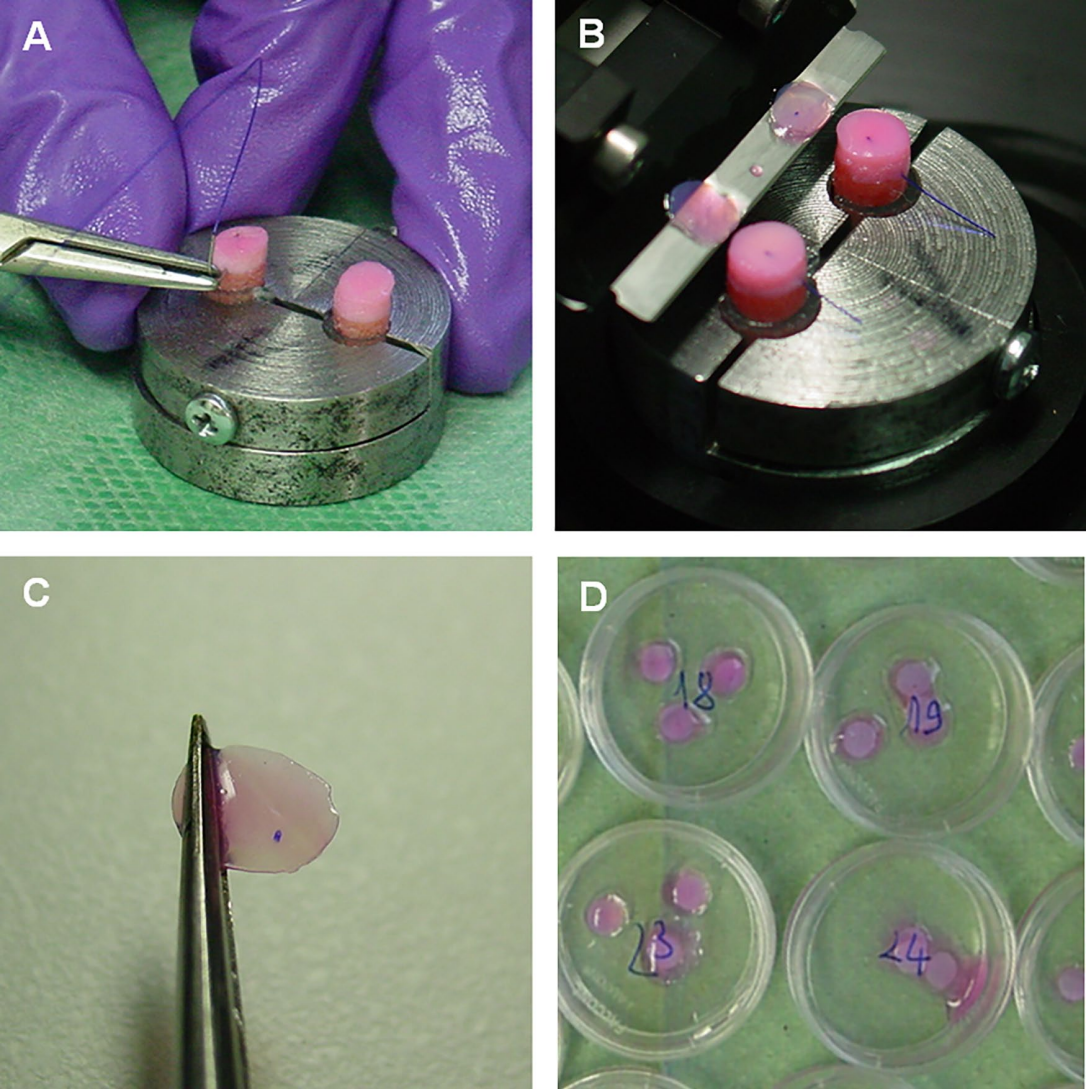
Demonstration of the trans-articular suture passage protocol: in-side out free-hand suture passing (**A**), transversal cartilage slicing with microtome (**B**), a representative 300 µm cartilage tissue slice with the thread *in situ* (**C**), Live/Dead molecular probes staining (**D**).

The cartilaginous part of the cylinders was transversely cut to 300 µm thick slices with a vibratory microtome (Oxford Vibratome model G; Oxford Laboratories, San Mateo, CA). The cartilage slices, including the thread, were stained with Live/Dead Viability/Cytotoxicity kit (Molecular Probes Inc., Eugene, OR). The kit contains 2 fluorogenic reagents: calcein-AM (Ca-AM) and ethidium homodimer-1 (EthD-1). Calcein is impermeable and therefore gets trapped by intact cell membranes. It emits green fluorescence at 517 nm (from 505 to 535 nm) when excited by blue light at 494 nm indicating that the cell has an intact membrane and esterase activity and is, therefore, considered viable. EthD-1 is impermeable to intact cell membranes but is able to diffuse through the porous membranes of dying or dead cells. This dye has a high affinity to nucleic acids and emits a bright red light at 617 nm (from 605 to 635 nm) when excited at 528 nm.^34^ The cartilage slices were incubated for 90 minutes at 37 °C with a solution of 1 µmol EthD-1 and 250 µmol Ca-AM diluted in saline (sodium chloride 0.9%; B. Braun, Melsungen, Germany). Five hundred microliters of each diluted dye was used and put into a 1.5 mL tube (Safe-Lock tubes) together with the cartilage slices. The contents of the tubes were protected from daylight during incubation, and washed before microscopic analysis. Using the staining protocol described, we were able to detect over 90% chondrocyte viability in every analyzed sample (see **Fig. 1** for protocol details).

### Confocal Laser Scanning Microscopy and Image Analysis

**Figure 2.**
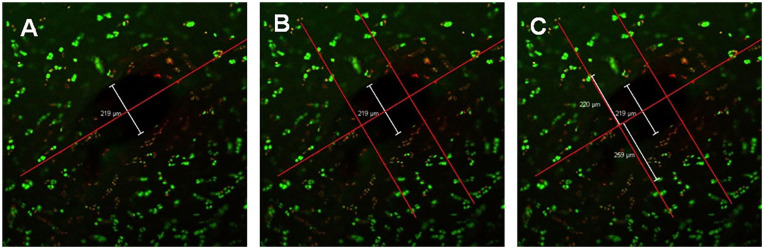
Image analysis of a transversal articular cartilage slice with an absorbable suture (PDS 4-0) after it had been stained with Live/Dead molecular probes and scanned under a confocal laser microscope. (**A**) The retained suture was identified in the image and a central longitudinal line was drawn to mark the suture passage canal (red line). A perpendicular line was drawn from the center to the maximal noncellular area on each side of the red line, as highlighted by white line. This represents the diameter of the missing articular tissue occupied by the suture material—the Black zone. (**B**) The region-of-interest for viable cells measurements was limited to one suture diameter around the suture mid-portion—the area between the 2 red perpendicular lines. (**C**) The Green zone diameter (the closest aggregated viable cells within the region-of-interest) was measured on each side of the central longitudinal line, as depicted by the 2 while lines. It represents the diameter of maximal chondrocyte injury by the suture passage and its in-tissue compressive forces.

The stained slices were analyzed by using an apochromatic objective lens (HCX PL APO 40 1.25 OIL) on a DM IRBE inverted microscope equipped with a 100 W Hg-lamp (Leica, Wetzlar, Germany). The confocal micrographs were scanned with a TCS SP2 CLSM equipped with a 488 nm argon/krypton laser line (Leica). The scans were taken at a 512 × 512 pixel resolution with the pinhole set at 1 airy unit. The location of the scan on each slice was arbitrarily chosen to avoid possible artifacts: 40 µm deep and at least 400 µm from the margins of the sample. The chosen location was captured by 7 images placed one above the other with the 10 µm in-between interval, giving an optical slice volume of 0.009 mm^3^. Confocal micrographs with green and red colored spots, Live and Dead cells, respectively, were analyzed using Leica Application Suite X (Leica MicroSystems, Wetzlar, Germany). The diameter of 2 zones of interest was measured in each slice (details in **Fig. 2**):

1. *Black zone*—the central zone without any cells, occupied by the remaining suture thread *in situ*. First a central longitudinal line was positioned along the suture passage canal occupied by the thread. The range of the Black zone was measured by summing the perpendicular distances from the central line on both sides to the maximum noncellular area. This measurement represents the *in situ* thread diameter.
2. *Green zone*—the diameter of the Green zone was determined from the minimum perpendicular distances from the central longitudinal line (on both sides) with aggregated Live (green) cells. This zone represents the maximum cell damage that occurred directly as a result of the suture but also due to the frictional and compressive forces along suture passage canal.

### Measurements of the Suture Needles and Threads Diameters

The true out-of-tissue diameter of the needles and threads were measured under the Olympus IX81F microscope with and Olympus UPlanFI 10× objective. The threads and needles measured came from the same manufacturing batch as the materials used in the viability experiment. The images were recorded with the Olympus DP71 camera and by using Olympus cellD Life Science documentation software. The latter was also used for the analysis of the images (Olympus Corporation, Tokyo, Japan). The needles were measured at the widest transversal diameter located at the mid-portion. Three short (2-3 cm) parts of the thread were cut out at defined lengths: just after the needle-thread junction, 15 cm and 30 cm away from it. The thread samples were moistened with saline solution before having been measured under the optical microscope. The measurements were repeated 4 times for each suture.

### Statistical Analysis

Numerical data are presented as averages with (SD). Diameters of the Black and Green zones are given first as absolute values, but also as Green/Black zone ratio (indicating the extent of maximum tissue damage by certain suture *in situ* diameter). The diameters of the Black and Green zones were additionally compared to the true thread diameters (Black zone to Thread diameter ratio, and Green zone to Thread diameter ratio). One-way ANOVA followed by a Tukey HSD posttest was used to test for significant differences between all 5 suture types. Additionally, a general linear model was implemented to test for possible influences of needle diameter, suture diameter, and suturing material toward the Black and Green zones. Statistical analysis was performed with statistical software IBM SPSS Statistics 23 (IBM Corp, Armonk, NY). A level of significance in all tests was set at *P* < 0.05.

## Results

Twenty-eight out of 40 prepared and scanned samples were eligible for image analysis. The Black zone diameters extended from 144 (28) µm (Vicryl 5-0 RB1+) to 219 (12) µm (PDS 4-0 RB1) (*P* < 0.001), while the Green zone diameters reached from 282 (35) µm (Vicryl 5-0 RB2) to 487 (67) µm (PDS 4-0 RB10 (*P* < 0.001). The ratios between Green to Black zone seemed quite constant, ranging from 1.87 (0.41) (PDS 5-0 RB2) to 2.24 (0.38) (PDS 4-0 RB1) (*P* = 0.767). Optical measurements of needle and thread diameters were exact and showed minimal standard deviations. Needle diameters were in the range from 287 (6) µm (Vicryl 5-0 RB2) to 478 (4) µm (Vicryl 5-0 RB1+), while thread diameters extended from 171 (2) µm (PDS 5-0 RB2) to 235 (30) µm (Vicryl 4-0 RB1+). Wider Black zone diameters were related to the thicker threads and to the PDS suturing material, but not to the needle diameters (*R*^2^ = 0.623, *P* < 0.001). The ratios between in-tissue (i.e., Black zone) and out-of-tissue thread diameters were in general larger for PDS (0.99-1.00) than for Vicryl (0.72-0.83) sutures. Similar trend was revealed for Green zone to thread ratios: PDS sutures 1.86 to 2.19, Vicryl sutures 1.39 to 1.60. The details are presented in **Tables 1** and **2**.

**Table 1.** The Extent of Acute Cartilage Injury on Viable Osteochondral Explants and the True Diameters of 5 Different Trans-Articular Sutures.

Suture (*n*)	Black Zone (µm)	Green Zone (µm)	Green to Black Zone (ratio)	Needle Diameter (µm)	Thread Diameter (µm)	Black Zone to Thread Diameter (Ratio)	Green Zone to Thread Diameter (Ratio)
PDS 4-0 RB1 (6)	219 (12)	487 (67)	2.24 (0.38)	460 (2)	222 (4)	0.99 (0.06)	2.19 (0.30)
PDS 5-0 RB2 (7)	171 (8)	318 (61)	1.87 (0.41)	373 (10)	171 (2)	1.00 (0.05)	1.86 (0.36)
Vicryl 4-0 RB1+ (7)	195 (22)	376 (60)	2.02 (0.36)	476 (4)	235 (30)	0.83 (0.09)	1.60 (0.26)
Vicryl 5-0 RB1+ (4)	145 (3)	290 (78)	2.00 (0.55)	478 (4)	190 (8)	0.76 (0.02)	1.53 (0.41)
Vicryl 5-0 RB2 (4)	144 (28)	282 (35)	2.05 (0.64)	287 (6)	203 (19)	0.72 (0.14)	1.39 (0.17)
	*P* < 0.001	*P* < 0.001	*P* = 0.767			*P* < 0.001	*P* = 0.006

**Table 2.** The General Linear Model Statistics Output.

Dependent Variable	GLM Sig.	*R* ^2^	Predictors	B	*P*	CI Lower	CI Upper
Black zone	<0.001	0.734	Suture material (PDS vs. Vicryl)	−53.862	0.000	−70.392	−37.332
			Needle diameter (µm)	−0.002	0.974	−0.130	0.126
			Thread diameter (µm)	1.190	0.000	0.809	1.570
Green zone	<0.001	0.623	Suture material (PDS vs. Vicryl)	−119.436	0.000	−176.105	−62.766
			Needle diameter (µm)	0.180	0.384	−0.241	0.600
			Thread diameter (µm)	2.532	0.001	1.210	3.854

CI = confidence interval.

## Discussion

The most important findings of this laboratory study on viable osteochondral explants evaluating the acute effect of different trans-articular sutures were the following: (a) the diameter of acute cartilage injury induced by the trans-articular sutures is about twice the thread thickness inside of the tissue; (b) less compressible monofilament PDS sutures seem to induce wider tissue injury than their softer braided Vicryl counterparts; (c) needle diameter did not correlate with the extent of acute cartilage injury. It seems that the impact of trans-articular sutures is caused directly by the suture thread, but also indirectly by expansive and frictional forces in the surrounding tissue.

Trans-articular sutures remain an important back-up for the fixation of different cartilage repair scaffolds and transplants in critical joint locations.^35,36^ Every surgeon needs to be aware of the potential iatrogenic chondral injury resulting from the usage of the fixation material. This is why it is recommended that the thinnest possible suture with sufficient holding properties is used. The impact of different suture materials on various soft-tissues has been extensively studied in some surgical fields, such as ENT, maxillofacial, plastic, obstetric, and thoracic surgery. A wide variety of macroscopic and histologic tissue reactions depending on studied materials were identified.^37-40^ Beauchamp *et al*. showed that tissue reactions persisted for longer when nonabsorbable sutures were used, and were related to suture material and diameter, suggesting Vicryl 8-0 to 10-0 as optimal in reconstructive tubal surgery.^37^ Other studies, directly comparing Vicryl to PDS, concluded that PDS was not only associated with a less intense acute and chronic inflammatory reaction, but also with lower adhesion formation.^38,40^ However, the research on the impact sutures have on the tissue in cartilage reparative and regenerative surgery is rather scarce. To the best of our knowledge, an animal study on goats by Hunziker and Stähli is the only one directly addressing problems resulting from the use of trans-articular cartilage sutures. The authors described a progressive loss of chondrocytes in the peri-sutural area of 200 µm. This chondrocyte loss was reminisced to the early stages of osteoarthritis.^26^ Some evidence related to the safety of trans-articular sutures can also be extrapolated also from the studies on menisci. Fibro-cartilage shares similar biological and biomechanical properties to the hyaline articular tissue. Yasunaga *et al*. studied the effect of different suture materials on meniscal and surrounding cartilage tissue in dogs’ knees, and they concluded there was a greater inflammatory reaction of the meniscal tissue to absorbable than to nonabsorbable suture materials. Additionally, the tissue did not regenerate after the suturing material was absorbed, but was only replaced with a reparative tissue, that is, the newly formed collagenous fibers were clearly different from normal meniscal tissue.^41^

Our study tested the acute effects of different suturing materials used in trans-articular sutures in cartilage restoration procedures. The idea was to directly compare the combinations of needles and threads, and provide surgeons with an evidence as to which suturing material should be preferred. We have employed *ex vivo* model with postmortem human osteochondral material combined with Live/Dead staining, which has been proven in many of our previous studies.^28-30,42^ Huntley *et al*. also used similar methodology when studying mosaicplasty coring tools. They concluded that the more the articular cartilage tissue was compressed during grafting, the larger the marginal zone of chondrocyte death is expected.^43^ This aligns with our observations, as Green to Black ratio (the ratio between living cartilage cells and the missing tissue) remained constantly twice as large as the missing tissue (i.e., Black zone) itself, which we attribute to the mass effect of the suture on the surrounding cartilage tissue. Osteochondral explants in our study protocol were fixed only at the bone level; therefore, cartilage was able to expand around the suture passage canal slightly more than in a contained articular surface. Referring to the study by Nabavi-Tabrizi *et al*. on osteochondral allograft transfer, we may speculate that the acute injury in natural conditions would be somewhat more intensive.^44^ Surprisingly, the needle diameters were found not to be directly related to the acute cartilage injury in our experiment. Cartilage as compressive viscoelastic tissue appears to be resistant to a transient passage of and atraumatic suture needle. It is the thread that remains in the tissue that causes both a direct (Black zone) and an indirect compressive chondrocyte injury (Green zone). The extent of direct injury of less compressive polydioxanone sutures was equivalent to their out-of-tissue diameters, whereas softer braided polyglactin was about 20% less voluminous inside of cartilage than out of it. A similar trend was observed in the diameter of indirect chondrocyte injury (Green zone), which was approximately double the out-of-tissue diameter of polydioxanone, but only about 1.5-fold of the diameter in polyglactin sutures. Artefacts by cartilage transversal cutting before confocal laser scanning microscopy, could not be avoided entirely. However, we would expect a more random pattern of suture deformity, if the latter had a meaningful impact.

One limitation of our study was the relatively small sample size of each needle suture combination used. This makes comparing the impact of different needle types on chondrocyte damage difficult, but nevertheless we managed to show significant differences in spite of such marginal sample sizes. The subexperiment for the estimation of the true out-of-tissue diameter of the needles and threads was conducted on the suturing material from the same manufacturing batch, but not with exactly the same sutures that was used for chondrocyte viability measurements. However, the measured true diameters were identical with minimal SD; therefore, a possible bias from this aspect was negligible. Another limitation was the technique used to determine Live/Dead chondrocytes in our study. The method of using fluorescent green and red Live/Dead probes had been questioned in previous studies as it was thought that it might not accurately show the real extent of cartilage tissue damage.^45^ With that in mind, we encourage further studies of this area, based on bigger sample sizes and leveraging different suture needle combinations—with a possible addition of growth factors to find the least injurious material for cartilage sutures in the future.^46^

## Conclusions

The width of acute cartilage injury resulting from usage of the trans-articular sutures is about twice the diameter of suture thread inside the tissue, suggesting that expansive and frictional forces around the suture passage further increase tissue damage. Less compressible monofilament PDS sutures seem to induce wider tissue injury than their softer braided Vicryl counterparts. There was no correlation found between needle diameter and the extent of acute cartilage injury.
